# Why Children's Hospitals Are Unique and So Essential

**DOI:** 10.3389/fped.2019.00305

**Published:** 2019-07-23

**Authors:** Georges Casimir

**Affiliations:** Department of Pediatric Pulmonology, Queen Fabiola Children's University Hospital, Université Libre de Bruxelles, Brussels, Belgium

**Keywords:** children, hospitals, multidisciplinary team, prognosis, teaching, research

## Abstract

Children's hospitals were created in the nineteenth century, first in France and then in England. They were designed to provide optimal care to infants, children, and adolescents in a specific environment where parents were admitted and where the special needs of children were catered for. No other system currently offers so many advantages as the multidisciplinary teams with their pediatricians, surgeons, anesthetists, intensive care specialists, and all the allied health professionals who can add their knowledge to the quality of care. From the beginning, they played a major role in caring for socially disadvantaged children. They brought together more than 95% of tertiary care, including cancer care and organ transplantations. They represent the best blend for the study of pediatric medicine and physiology, for high-level preventive medicine, and for research in all fields of pediatrics. This probably explains why they have developed everywhere around the world. This article explains the paramount importance of children's hospitals for providing safe and effective multidisciplinary pediatric care.

## Introduction

There are more than 250 children's hospitals (CH) in the US (about 1 for every 20 of adults), providing over 95% of transplantations, cancer programs, and tertiary cares (1). These hospitals also play a major role for disadvantaged children. This way of organizing pediatric care is now firmly established as the standard for providing the best tertiary care programs, in industrialized but also in developing countries (2, 3). This indicates that pediatric departments in general hospitals (GH), including university hospitals, are not the common approach for care to children in the world, since CH offer patients, and their families a multidisciplinary alternative centered on the child. It is also a matter of critical mass: only CH can provide programs where surgeons, anesthetists, radiologists, and allied health professionals are sufficiently numerous, trained, and motivated to obtain the best results (4).

## History

The first hospital dedicated to children was created in Paris (1801). It was called *Hôpital Necker-Enfants Malades*, after Suzanne Necker, wife of the finance minister of Louis XVI. It was a *public* hospital, founded to care for children of both sexes under 15 years of age. The hospital had gardens and 250 beds. Grouping children together clearly promoted the study of pediatric diseases, development, and nutrition. Children were divided according to their sex and disease into rooms of 30 to 40 beds. They had separate walkways, planted with linden trees. Proper hygiene was observed. The number of beds rose above 600 in 1850. It was in this hospital that Laennec invented the stethoscope (1816) and that Chaillou and Martin successfully used the diphtheria antitoxin for the first time. This hospital also witnessed a series of great scientific achievements: first kidney transplantation in France by Hamburger (1952), first bone marrow transplantation for immunodeficiency by Griscelli (1972), first gene therapy by Alain Fischer (1999), and first stent and valvular prosthesis in pediatric cardiology (2000).

Great Ormond Street Hospital for Children was created in London (1852). It opened with 10 beds and was the first hospital in Britain to offer dedicated care to children. In this hospital, Sir Thomas Smith was the first surgeon to attempt antiseptic surgery in 1875. He also invented a device for administering chloroform to treat very young children. Lady Superintendent Catherine Jane Wood introduced a pediatric training program for the specialization of nurses. Mildred Creak (1898–1993) was the founder of child psychiatry in Britain. Creak championed collaboration between pediatricians and psychiatrists. She also increased visiting hours for parents, encouraging appreciation of children's emotional needs and helping parents in their distress.

In the US, Dr. Francis Henry Brown created in 1869 the Boston Children's Hospital, one of the most famous in the world. A civil war surgeon, Brown traveled to Europe in 1867 to study the pioneering specialized approach to treating children. Brown was impressed with the treatments he witnessed and wanted to bring that level of care to Boston. He opened a 20-bed facility in a small townhouse. The hospital became affiliated with Harvard in 1903. Today, the Boston CH is a 404-bed comprehensive center, with remarkable levels of excellence.

## Definition and Characteristics of CH

CH is usually learning centers affiliated with university hospitals, localized in important urban centers. During the 20th century, pediatric medicine became completely separate from adult medicine. But pediatric hospitalization is heterogeneous, even in industrialized countries, and local GH, with only a section dedicated to pediatrics, with other adult specialities in the same building, remain usual. This situation requires narrow links with the regional multispecialized pediatric hospital to organize transfers of unstable children and regular training with general pediatricians and/or adult specialists. Four types of CH were identified in Europe according to their structure and links (1) (cf. Supplementary Table). Stand alone and university CH are tertiary structures from a network characterized by reference and concentration of patients needing highly specialized cares or diagnostic procedures. Cooperation between the different levels of cares (CH, secondary pediatric departments, rehabilitation centers, and palliative care hospices) must be determined by care paths. They also play a fundamental role in educating care providers, for example by promoting medical research in the field. CH are also important for future adult specialists or laboratory doctors, because they offer possibility to observe pediatric practice and hospitalization.

Unfortunately, there are no database that would allow the validation of all the underlying reasons for the different proportion of CH and hospital beds per 1 million child population. Merging the need for healthcare efficiency with socially compatible physical spaces to children and their caregivers is now becoming the leading approach in projects devoted to building new CH.

The healthcare needs of European and American children are evolving as a result of changes in the diseases, disabilities, and social factors that affect their lives. Infectious diseases have become easier to prevent and cure, whereas non-communicable diseases now dominate pediatric practice. Rare disorders like cancer, metabolic diseases, and some neonatal or complex conditions need highly specialized care in centers, while more common and less severe conditions are usually treated in outpatient care and community practice to enable children and their families to live as normally as possible (5).

Today rare diseases are studied in CH, from the beginning of their history. This offers fertile ground for investigation to the teams.

CH have consistently shown greater development in terms of volume, programs of care, multidisciplinary approaches and the concept of humanizing the hospital than have pediatric departments in GH. Other medical specialties such as surgery, anesthetics, radiology, otorhinolaryngology, ophthalmology, and dermatology have been developed within programs of care where the child is the focal point bringing physicians, nurses, and allied health professionals together. CH usually build research institutes (6), playing a major role in the mutual enrichment of medicine and laboratories with a multiplier effect on medical creativity. The research institutes in pediatrics are closely linked to teaching and training, but also to cares.

CH are not just buildings. Besides caring and providing for sick children in emergency and chronic settings, they contribute in essential ways to primary care and wellness, to all kinds of prevention including child abuse, and to health fairs and in-school health services. The unique role of CH in the prevention of social exclusion and violence is determinant for the present and future.

In the past, obstetricians expressed their desire to benefit from pediatricians to care for and save the infants they helped to deliver, while adult surgeons needed pediatricians for the postoperative care of children. Difficult and time-consuming, pediatric cares continue to be reimbursed at the lowest levels in many countries, even in industrialized ones, showing the lack of consideration for children and their caregivers. In GH, tensions between physicians caring for adults and those caring for children remain common, especially in departments where they should be working together toward a shared objective that ought not to be undermined by frivolous power struggles. Even today, departments such as neonatal, emergency and pediatric intensive care units, which legally should be separate from those of adults, are frequently re-combined in terms of their administrative management for obvious budgetary or strategic reasons with no bearing on pediatricians.

## Cooperation Between CH and Other Structures, Child-Centered Care

CH are designed with children in mind. They have specialists, allied health professionals and technology for children not found in other hospitals. Depending on the development and physiology of the child, the size of machines and the specific procedure for administering a drug can make different equipment or specific expertise necessary.

In the US, CH perform 97% of all organ transplantations and 90% of all pediatric cancer care. However, as children are less often ill and hospitalized than adults, only 1 hospital in 20 is a CH. There are about 250 CH in the US which play a much greater regional role than GH, especially for rare diseases, for which multidisciplinary follow-up is organized.

## Mother and Child Hospitals

CH are now becoming “mother and child” hospitals, because high-risk pregnancies have made the proximity of the CH necessary to providing proper care to the fragile child, but also to the mother, transported to the maternity before delivery. Grouping maternity units together to reach a sufficient number of births is critical for reducing both infant and mother mortality rates. The number of 800 deliveries appears to be a strong determinant of the prevention of risks for mother and child (7, 8), improving quality and prognosis of neonatal surgery. Neonatal malformations that require quick, well-controlled surgical interventions can only be corrected by teams with a critical mass in terms of number of cases and health professionals. A better organization at national (or regional) level tends to minimize the burden of surgical limitations faced by professionals who are few in number. Exposure to a higher number of cases typically reduces the rate of complications.

High-risk deliveries represent only 6% of all births. Referring the mother to a high-risk maternity prior to delivery safeguards the activity and funding of the referring center, usually not equipped and financially resourced. Grouping obstetricians from different maternities and even different universities together in one building near a children's hospital can aid the creation of centers of excellence. It is probable that, in the future, demands of families or mother and specific child situations will transform some pediatric hospitalizations, requiring new organizations.

## Neonatal Intensive Care (NICU), Pediatric Intensive Care (PICU), Pediatric Surgery and Intermediate Care Wards

Over the past 5 decades, new surgical techniques in neonatal critical care have totally modified the morbidity and mortality of many diseases, including congenital diaphragmatic hernia, intestinal atresia, tracheoesophageal fistula and omphalocele. Congenital heart diseases have become totally correctable with open-heart surgery, and effective combination therapy of many cancers has allowed pediatric surgery (PS) to play an essential role in improving the quality of care. In the 1950s, the role of PS was still limited, because palliative procedures were the only treatment for many malformations, with 85% mortality within 24 h for congenital diaphragmatic hernia. There were no neonatologists and no one was interested in the specific problem of prematurity and newborn physiology (9). However, PS later became a better-defined interest area within general surgery. Pediatric cancer surgery then became individualized, with unusual malignancies occurring only in childhood and requiring careful multidisciplinary management by a team of pediatric surgeons, and oncologists. Some pathologic conditions are only seen in children, such as pyloric stenosis, intussusception, and midgut volvulus. Splenectomy was increasingly performed in children suffering from spherocytosis. If congenital abnormalities could be corrected, most of these children would lead normal, productive lives (10).

This specialty was not yet recognized in the majority of European countries, except in CH where rare and complicated cases would be centralized to improve surgery and intensive care with a nutritional strategy. The surgical section of the American Academy of Pediatrics played a major role in establishing specific training in PS and finally board certification in general surgery with 2 additional years of specialized training in PS. In 1970, the American Pediatric Surgical Association (11) was finally established, and PS was officially recognized in the US in 1972. The sole chair in PS before this recognition had been that of the famous Professor Ladd in Boston. But surgeons would be nothing without well-trained anesthetists who ensure the exceptional quality of the management. That is why CH are so well-suited to enabling collaboration between all subspecialties.

Coelioscopic and robotic surgeries are now rapidly developing, with miniaturization of instruments that plays a major role in prognosis.

## Characteristics of Pediatric Hospitalization

Nearly one in six discharges from American hospitals in 2012 involved children aged 17 years and younger (12). The majority were infants, including newborns. Even if the rate of hospitalization decreased annually by 0.6% among infants and by 0.9% among children aged 1–17 years, the average annual growth in mean hospital costs per stay during the same period was 6.7% for infants, and 6.4% for children aged 1–17 years, more than three *times* the rate of cost growth of any other age group. The volume of care for chronic conditions is one of the reasons for this growth (13). However, there is always a time lag between the reality of the situation and the time politicians take to react.

Excluding neonatal stays, adults aged 18–44 years had a hospitalization rate that was 1.7 times higher than that of children. The majority of hospital stays among children were for newborns. These neonatal stays excluded, mean hospital costs were higher for children than for adults. However, nearly three quarters of hospital stays for children and more than half of all hospital costs for children were for newborns and infants. Respiratory conditions (pneumonia, bronchitis, and asthma) were the most frequent reasons for hospitalization, followed by digestive conditions and then nervous system and mental disorders. Appendicitis was among the most common reasons for hospital stays among both children and adults. CH group together multidisciplinary teams for rare and complex diseases as well as for the complex management of newborns with malformations and/or metabolic disorders. That is why their funding needs to be specifically considered (14).

The profile of pediatric diseases is gradually changing: many children now survive extreme prematurity; the number of children with chronic diseases is increasing; the obesity rate is growing; more children and adolescents are suffering from mental health problems; pediatric cancers and malformations are better cared for; and intensive care techniques have improved. Funding needs to adapt to these circumstances.

## The Pediatric Environment

Patients of any age, but especially children, require healthcare that is focused on their unique needs from start to finish, and that involves their family, particularly their parents. This concept can be traced back to Hippocrates. Its funding should not be influenced by financial considerations, and it is best implemented when delivered in a child-centered care environment. Children require more time and specificities for their care (ultrasound and small serum volumes for lab investigations), close monitoring of their parameters, personalized medication and specially trained care providers who are compassionate. CH offer the right conditions (kindergarten, school, psychology team, ergo, and speech therapists) to enable high-quality treatment to coexist with a philosophy that fully aims at humanizing healthcare. The success of the model is demonstrated in the real world by the extraordinary development of CH in both industrialized and developing countries despite the financial and organizational issues.

In CH, every detail is thought out to optimize the care, upbringing and well-being of the child. The school plays a central role in CH, as do the parents and families. The attractiveness of environment and color scheme is also important, and are employed to reflect their dreams, and carefreeness. Whereas, CH are built according to these basic principles, it is not always the case for GH containing pediatric departments, where designing a pediatric environment is not necessarily easy or financially feasible. Although legislation on the rights of patients and the charter for children in hospital oblige authorities to give thought to the pediatric environment in the departments, this does not apply to the other departments of the GH, such as surgery, anesthesiology, radiology, and otorhinolaryngology, where adults and children are still frequently mixed. In practice, all hospitals try to make their pediatric wings fun and kid-friendly, but in CH the entire building is like this.

## Market Relevance of CH, Future Role and Economic Dilemne

But CH are also essential for children from poorer backgrounds. In the US, half of all the care provided in CH goes to disadvantaged children, and 6% are medically complex, requiring ongoing care for serious, long-term conditions. If these hospitals did not exist, the care that these children receive would be random, and uncertain. Ensuring high-quality care and maintaining a robust pediatric system for delivering that care require that knowledge, and technical procedures be well-paid. The fees are usually not high enough in pediatrics. If professionals are not properly paid, it is impossible to retain staff and encourage an adequate influx of newly trained pediatric providers (15). A focus on improving the funding of healthcare for children and adolescents will likely result in a high long-term return on investment for society as a result of the prevention and early detection.

In the past, CH were under less financial pressure than GH, and they faced fewer government requirements and penalties, but with increased importance of health insurance, heightened consumerism and fierce competition from non-CH, and other pediatric providers, they must now take action to communicate their relevance to strategic partners. With their multidisciplinary teams, expert staff and facilities, CH may be at a unique advantage in demonstrating their particular relevance. However, like other institutions, their overall care costs may be higher than their community counterparts. Thus, they must be creative when demonstrating their value in the delivery of safe and efficient care.

The decline in inpatient discharge rates in CH is expected to be 4% (compared to 10% in non-children's hospitals). This must be considered in the strategy alongside the increase in outpatient services, which are projected to grow by 7%. Hence considering new outpatient buildings, and possibly connecting these to research facilities, could help CH augment their attractiveness, with continuous training and education of staff, as well as quality initiatives. Partnerships with general hospitals for the transition between childhood and adulthood in chronic diseases is one of the challenges for both hospitals.

CH must offer quick access to consumers and partner physicians to consultations with pediatric subspecialists, for example neurologists and pulmonologists. Even if costs are higher in CH, hospitals must try to optimize their costs by reducing unnecessary hospital stays, and involving family members in the care program.

However, it is still necessary to defend this model of care, which continues to be questioned on financial grounds or because organizational factors favor adult medicine. This is especially true since the place of women and children in our society is not yet what it should be.

## Author Contributions

GC conceptualized the paper, drafted the manuscript, and approved the final manuscript as submitted.

### Conflict of Interest Statement

The author declares that the research was conducted in the absence of any commercial or financial relationships that could be construed as a potential conflict of interest.

## Abbreviations

CH: children's hospitals
GH: general hospitals
PS: pediatric surgery
US: United States.
