# Supplementary material for: Why Children's Hospitals Are Unique and So Essential
Source: Front Pediatr. 2019 Jul 23;7:305. doi: 10.3389/fped.2019.00305 (PMC6664869; doi:10.3389/fped.2019.00305)
Supplement: Supplementary file 1 [file Data_Sheet_1.PDF]

Table

Types of Children's hospitals in Europe

	General hospitals with pediatric departments	Stand alone independant chidren's hospitals	University children's hospitals	Highly specialized pediatric centers of competence and mother and child centers
Day clinic	+	+	+	+
Neonatal intensive care unit	+	+	+	+
Pediatric intensive care unit	+/-	+	+	+/-
Frequence on 46 countries	?	> 80%	?	?
Pediatric imaging department	-*	+	+	+
Pediatric surgery department	-*	+	+	+

***usually adult radiologist/surgeon working in pediatrics**

Source : European Association Pediatric survey 2009
